# Evolution Law of Contact Force Chain Network Structure of Geotechnical Granular Materials Under Unloading Stress Paths

**DOI:** 10.3390/ma19061158

**Published:** 2026-03-16

**Authors:** Gang Wei, Jinshan Tong, Luju Liang, Changfan Yu, Guohui Feng, Xinjiang Wei

**Affiliations:** 1 Department of Civil Engineering, Hangzhou City University, Hangzhou 310015, China; weig@hzcu.edu.cn (G.W.); 2230302005@stu.hzcu.edu.cn (J.T.); 2240302011@stu.hzcu.edu.cn (C.Y.); ghfeng@hzcu.edu.cn (G.F.); weixj@hzcu.edu.cn (X.W.); 2 School of Civil Engineering and Architecture, Anhui University of Science & Technology, Huainan 232001, China; 3 School of Architecture and Engineering, Zhejiang University, Hangzhou 310058, China

**Keywords:** granular matter, persistent homology, force chains, DEM, unloading stress path

## Abstract

Granular materials exhibit complex mechanical behaviors during unloading, yet the underlying micro- and meso-scale mechanisms remain unclear. This study employs a discrete element method to simulate a series of triaxial tests on sand and pebble specimens with varying initial densities under different unloading stress paths. While dense specimens demonstrate strain softening and dilatancy, loose samples exhibit shear contraction. To quantify the underlying fabric evolution, persistent homology (PH) theory is adopted to analyze the particle contact force networks. The results reveal that the average strength of this network correlates strongly with the macroscopic stress–strain response. For dense samples, network strength rapidly increases to a peak coinciding with the deviatoric stress maximum, then gradually decreases with further shear. Crucially, this evolution is path-dependent: the average contact force network strength increases approximately 20% more during unloading in the minor principal stress direction compared to unloading in the major principal stress direction. This quantitative analysis of force chain degradation provides a mechanistic explanation for the observed strain softening, highlighting the dominant role of the unloading stress path. In contrast, loose specimens, which initially lack an obvious force chain network, show negligible microstructural evolution during unloading.

## 1. Introduction

Geotechnical granular materials, such as sand, pebbles, stockpiles, coarse-grained soils, etc., are basic materials in the field of civil engineering. Their shear strength and deformation damage characteristics are highly related to the safety of earth dams, shield tunnels, foundation pits and other projects [1,2,3]. In underground engineering construction, engineering disturbance will cause the ground soil to experience special unloading stress paths. Taking shield tunneling as an example, shield tunneling will induce unloading deformation of overlying soil in the direction of large principal stresses, while in foundation pit engineering, excavation will cause unloading deformation of the surrounding soil in the direction of small principal stresses, which can induce geotechnical disasters such as instability of the shield tunnel excavation surface, failure of the foundation pit support structure, and even ground subsidence in severe cases [4,5,6]. However, for a long time, the strength index of the soil body determined by a conventional triaxial compression test was often used in the deformation calculation of the geotechnical body around the shield tunneling project and foundation pit project, which is actually very different from the unloading stress path experienced by the geotechnical body in the process of shield tunneling and foundation pit excavation. The mechanical parameters of surrounding soil under an unloading state are also different than under a loading state as simulated in traditional laboratory triaxial tests. Since the strength and deformation characteristics of geotechnical granular materials have significant stress–path correlation, the study of the stress–strain relationship of geotechnical granular materials under the unloading stress path and its deformation mechanism is still of great significance and application value for disaster prevention and mitigation of underground engineering as well as for the safe operation of underground infrastructure.

At present, scholars study the mechanical properties of geotechnical granular materials under unloading stress paths based on model tests and numerical simulations. For example, Li et al. [7] conducted triaxial high-pressure unloading tests on sandy soils in deep strata and explored the internal structure evolution by a CT device. The results show that during the unloading process, the soils began to show lateral relaxation, particle density decreased, and pore space increased, but the directional arrangement of particles and pores in the transverse section remained unchanged, and this directionality was not destroyed until the failure of the soil sample. Li et al. [8,9] conducted triaxial loading and unloading tests on sandy soils with different densities, shear contraction and shear dilation were found in dense sand under loading and unloading test, respectively, while only shear contraction is observed in both loading and unloading test of loose sand. However, the unloading deformation–stabilization process of a geotechnical body is a physical process with multi-scale behavioral patterns, and it is difficult to understand the mechanical mechanism behind this complex unloading mechanical behavior only from macroscopic-level tests. The evolution of the microstructure and mesoscopic organization of the dispersed geotechnical body in the unloading process is of great significance for better understanding the intrinsic mechanism of the constitutive model of granular materials and the structural origin of the deformation instability of soil mass. Therefore, it is necessary to use suitable methods in microscale and mesoscale analysis to further clarify the multi-scale characteristics of mechanical properties of geotechnical materials [10,11,12,13].

The key to study the deformation and instability process of a geotechnical body under an unloading stress path is the destruction and reconstruction of the force chain network structure composed of particle contacts between soil particles under unloading, which is difficult to be quantitatively characterize by traditional soil mechanics theory and geotechnical engineering parameters. In recent years, the complex network theory [14,15] has been widely used in multidisciplinary fields such as transportation planning, internet, acoustic conduction, and viral propagation as an effective method to reveal and characterize the statistical properties of network systems and to measure these properties. Studies have shown that the contact force chain system of granular materials also has the typical small-world characteristics and scale-free nature of complex networks [16], and the basic parameters used to characterize the properties of the network system in a complex network theory can not only describe the change in the relative compactness of the soil under the action of the load, but also describe the non-equivalent state of the characteristics of the pore structure and the arrangement of particles under the same compactness [17,18,19,20]. Therefore, in recent years, the microscale analysis method based on the complex network theory has received more and more attention from researchers and scholars in the field of geotechnical engineering. A complex network theory, such as persistent homology (PH), is a fractal method that performs multi-scale analysis on a fractional complex network. Compared to other existing methods, PH can identify topological structures such as clusters, holes, and cavities of the contact force network in granular material. Therefore, it has been gradually applied to the contact force network analysis of geotechnical granular materials in the process of shear, compression, and vibration. Research results show that under different loading conditions, the contact force network structure of geotechnical particles will change greatly, which is manifested by the change in the topological parameters of the network (such as the degree distribution, clustering coefficient, force circle, average path length, etc.) [21,22,23], and thus has an important impact on the macro-deformation of the particle system. However, the analysis of the evolution of the contact force network structure in granular soils under different unloading stress paths has not been reported yet.

Aiming at mechanisms of the deformation instability process of geotechnical granular materials under unloading conditions, this study adopts the discrete element method to simulate the triaxial test of geotechnical granular materials under different unloading stress paths. Based on the persistent homology method, comparative analysis of the topology and structural characteristics of the complex contact force network system constituted by soil particle contacts in the unloading process are conducted, in order to explore the critical response of instability of granular materials from the initial state to the critical state under the framework of complex network theory, providing a new perspective for the understanding of macro-mechanical behaviors of the granular system under three-dimensional unloading conditions.

## 2. Methodology

### 2.1. Soil Particle Modeling

**Figure 1:**
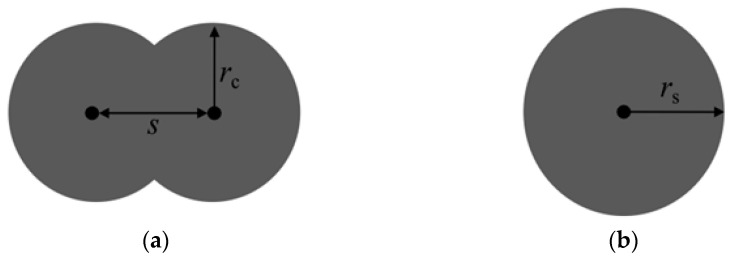
Discrete element numerical modeling: (**a**) sandy soil; (**b**) pebbles.

In this paper, the commercial discrete element software PFC3D (Version [6.0], developed by Itasca Consulting Group, Minneapolis, MN, USA) is used to carry out triaxial test simulations under different unloading stress paths of sand and pebble with two different grain sizes. As shown in Figure 1, in the discrete element model, the sand particles are simulated as a single spherical particle (ball), while the pebbles are simulated by clumped particles consisting of two overlapping spheres. Here, *r_s_* is the radius of the spherical sand particles. The particle size of the pebble particles is represented by the equivalent particle diameter (*d_s_*), defined as the diameter of the spherical particle with the same volume as the clumped particle. *r_c_* is the radius of the clumped particle, *s* is the center distance between the two clumped particles in the pebble particle. In this study, *r_c_/s* is 0.7.

**Figure 2:**
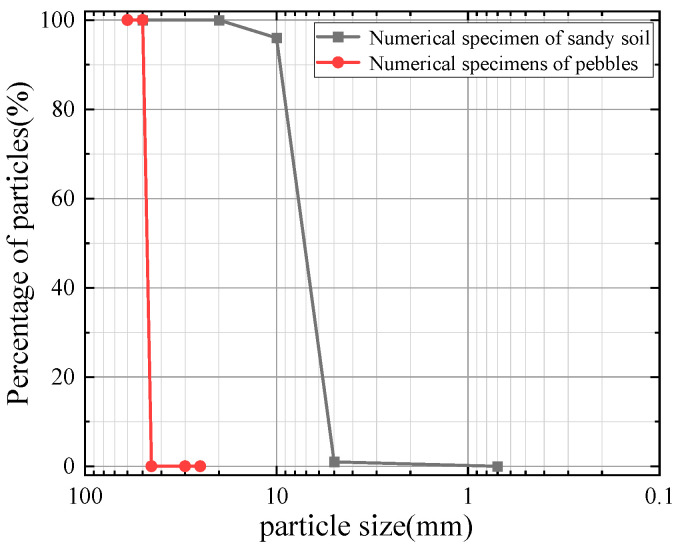
Particle size distribution analysis.

Grading curves of the particles in this study are presented in Figure 2. The particle size distribution used in the simulation is based on the soil material from Reference [24], where it was determined by sieve analysis. According to Reference [24], the numerical sample is generated to simulate the ISO standard sand [25] (medium grade) produced by Xiamen ISO Standard Sand Co., Ltd., Xiamen, China, with a particle size ranging from 0.5 mm to 1.0 mm, and the granite cobbles are sourced from a quarry in Baoding, Hebei Province, with particle sizes between 40 mm and 50 mm. In order to ensure the efficiency of the model calculation, the particle size of the sand particles is enlarged by 10 times for the simulation in this study [26,27]. The median particle size of sand particles *d*_50_ = 6.95 mm, gradation non-uniformity coefficient C_u_ = 1.21, the median particle size of pebble particles *d*_50_ = 63.2 mm, and the gradation non-uniformity coefficient C_u_ = 1.03.

### 2.2. Numerical Model for Triaxial Tests

A total of 24 simulations were designed considering different combinations of porosity (*n*), major principal stress (*σ*_1_), minor principal stress (*σ*_3_), and stress path. The general configuration of DEM simulations is summarized in Table 1. To generate the specimens (0.45 m × 0.45 m × 1.125 m) required for the triaxial test simulation, non-overlapping particles (about 15,000 particles for sand simulation and 5000 particles for pebble simulation) were first randomly generated in a larger cubic space. Then, the granular assembly was isotopically consolidated to *σ*_1_ = *σ*_2_ = *σ*_3_ = 0.1 MPa by six rigid walls through serve control. By setting different inter-particle friction coefficients during consolidation, granular specimens with different initial porosities can be obtained [28,29,30]. Coefficients of inter-particle friction during consolidation of sand were 0.01, 0.10, and 0.50 for the specimens with initial porosities of 0.35, 0.40, and 0.45, as seen in Table 1, and 0.10, 0.20, and 0.50 for pebble consolidation.

**Figure 3:**
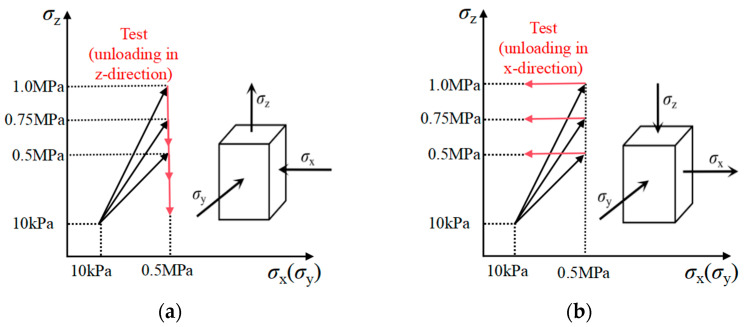
Stress paths of the triaxial numerical simulations: (**a**) stress path A; (**b**) stress path B.

Subsequently, after the model specimens were consolidated to the initial state of stresses required for the simulation, triaxial test simulations were conducted according to the stress paths as shown in Figure 3. During the simulation process, the unloading rates of both stress paths were set as 0.005 m/s and the time step was set as automatically calculated by the software so that the specimens can satisfy the quasi-static conditions. This can be checked in the simulation of the triaxial unloading test by stopping the simulation in the unloading process and observing that the load remains nearly constant. It is noted that a slower velocity would provide more stable results; however, it would lead to an unaffordable simulation due to the long computing time cost.

In the simulation of particle flow programs, reasonable calibration of the simulation parameters is crucial to ensure that the simulation results are consistent with the experimental data. The inter-particle behaviors simulation involved in this paper includes the contact between sand particles and the contact between pebble particles, all of which are carried out based on the linear elastic contact model. Specifically, the simulation parameters that need to be accurately calibrated include the particle linear contact modulus (*E_c_*), normal contact stiffness (*k_n_*), tangential contact stiffness (*k_s_*), and the friction coefficient (*f*). In this study, we refer to the results of large-scale triaxial tests on sandy pebble soils in reference [24], and define the sandy soil specimen with 0% stone content in the large-scale experiments as the sandy soil specimen in this paper, and the soil specimen with 100% stone content as the pebble specimen in this paper. All contact parameters were calibrated by comparing the numerical triaxial test results and the real triaxial test results in the literature.

**Figure 4:**
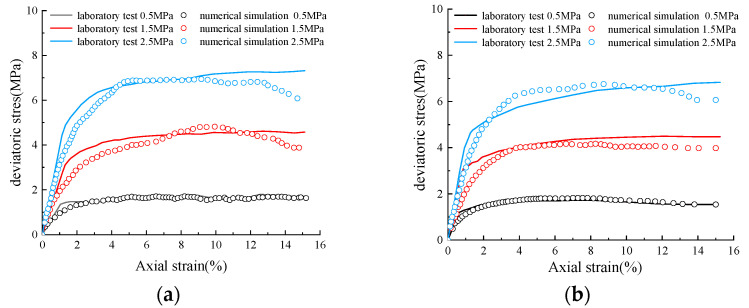
Comparison of indoor test and numerical simulation: (**a**) stress–strain curves for sandy soils; (**b**) stress–strain curves for pebbles.

Table 2 lists each fine-scale simulation parameter after calibration. A detailed comparison of the stress–strain curves obtained from numerical simulations with the indoor test data under different confining pressures (0.5 MPa, 1.5 MPa and 2.5 MPa) is shown in Figure 4. From Figure 4, it can be seen that the numerical simulation results basically coincide with the stress–strain curves obtained from the experiments, and the final determined parameters are coincident with the range of parameters that are widely adopted in relevant studies (e.g., [2,23,24,26]), which indicates that the relevant coefficients adopted in this study are reasonable.

## 3. Particle Contact Force Chain Network Analysis Method

In order to analyze granular materials’ behaviors from the perspective of complex network theory, the microstructure of granular materials should be abstracted into a topological network structure. In the discrete element simulation, the center of each particle can be regarded as a node vi (i = 1, …, N) in the topological space, and each pair of contacts between particles can be regarded as a pair of edges <*v_i_*, *v_j_*>, which can simplify the granular system into a complex network (CN) with topological properties. The particle contact network evolves continuously because the particle contact relationship between different particles is constantly adjusted during the triaxial test. The complex network constructed by this method is a weighted network due to the different contact forces between different particles. In order to quantitatively analyze the weighted network composed of inter-particle contacts at different moments during the simulation process, persistent homology (PH), a topological data analysis method [31,32], is adopted in this study. This method can be used to set up different contact force thresholds *θ* for the particle contact network, draw persistent homology diagrams, and then analyze the topological structure variation in the contact force network with contact force greater than the threshold value during the process of the threshold value’s continuous decrease. Therefore, it can quantitatively evaluate the number of characteristic structures (so-called force chains) in the particle contact force networks and the degree of strength of the characteristic structures during the triaxial simulation process.

**Figure 5:**
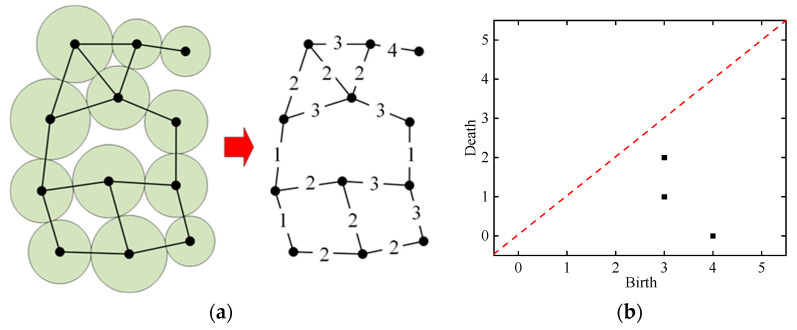
Analysis example of force chain network structure and continuous homology theory: (**a**) schematic of the particle contact force chain network; (**b**) continuous homography PD*β*_0_.

Taking the particle contact force network in Figure 5a as an example (in this example, the particle contact force magnitude is set artificially, whereas in the subsequent analyses in this paper, the particle contact force magnitude is obtained by discrete element numerical simulation), the simplest persistence diagrams of this force network are shown (called PD*β*_0_), which are mainly used to describe when the force chains in the contact force network experience “birth” and “death”.

When an inter-particle contacts not connected to other contacts appears in the force network as the contact force threshold decreases, it is characterized as a “birth” of a topological feature. With successive decreases in the contact force threshold, the newly emerging contacts in the force chain network will connect to this contact and form a force chain. When a newly emerging contact connects the force chain with another existing force chain, it is recognized as the “death” of the force chain. Therefore, in PD*β*_0_, each point (*b*, *d*) represents a component in the force network, and the magnitude of *b*–*d* can be interpreted as the notable degree of this component. As an example, in Figure 5, at a contact force threshold *θ* = 4, the first pair of inter-particle contacts occurs at the top of the force network, and thus this feature is represented in Figure 5b by a point with a horizontal coordinate b = 4. In Figure 5b, the two other points with the horizontal coordinate b = 3 characterize two sets of cluster structures that start to appear in the force network when the contact force threshold is reduced to 3. One set is located at the bottom of the force network in Figure 5a, and the other is located at the second layer from the top. Among them, the component located in the middle is connected to the component located at the top of the force chain network when the contact force threshold is decreased to *θ* = 2, and thus the vertical coordinate of the point characterizing this component in Figure 5b is *d* = 2. The component located at the bottom of the force network connects with other components when the contact force threshold is decreased to *θ* = 1, and thus the vertical coordinate of the corresponding point in Figure 5b is *d* = 1.

**Figure 6:**
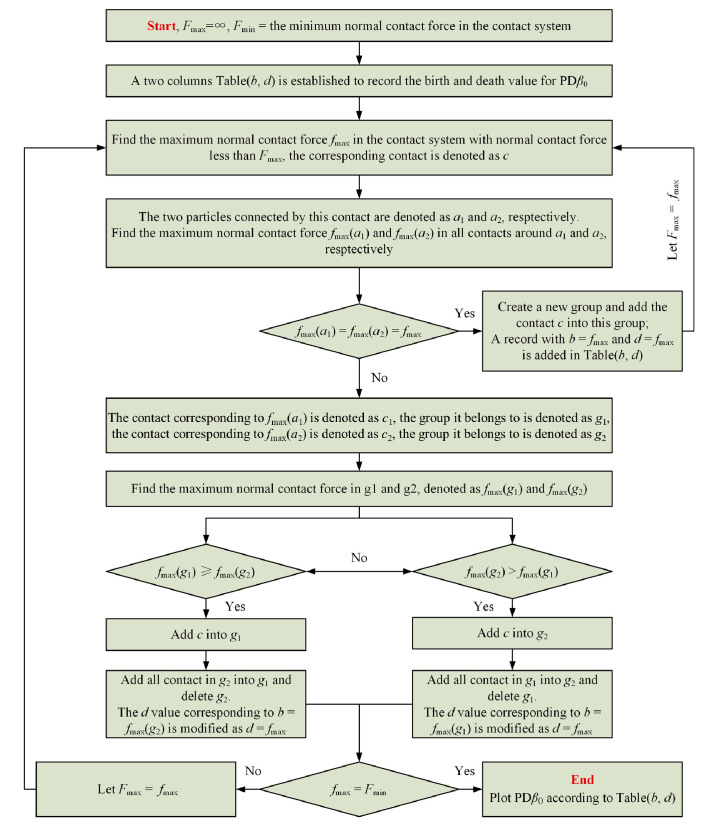
Continuous homology diagram PD*β*_0_ drawing program flow.

The main advantage of using PH to study the micromechanical behavior of granular materials is that it can quantitatively analyze when the topological features in the force network reach “birth” and “death” without artificially classifying the “strong contact force” and “weak contact force”, as the granular contact force threshold value is successively changed from the maximal contact force to zero. A more detailed presentation of this theory can be found in [33,34]. In this study, in order to analyze the force network structure of granular specimens during the discrete element simulation of triaxial unloading test through PH, a program is written to draw the persistence diagram PD*β*_0_*,* and the basic flow of the program is shown in Figure 6.

## 4. Results and Discussion

### 4.1. Stress–Strain Behaviors

**Figure 7:**
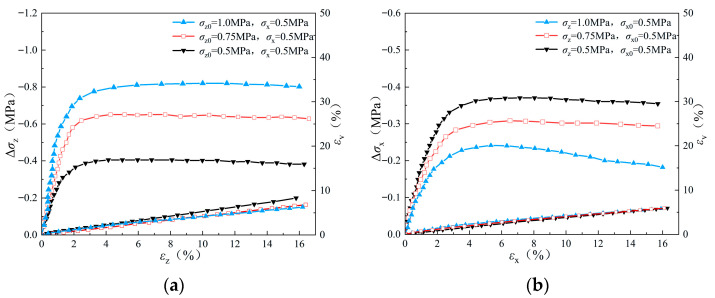
The evolution of stress–strain with axial strain during unloading of sand sample (*e* = 0.47): (**a**) stress path A; (**b**) stress path B.

The macroscopic stress–strain behaviors of the sand specimen during triaxial loading and unloading simulations are shown in Figure 7. During the unloading process under stress path A, the stress increment is observed to rise gradually and reach the peak value with the increase in axial strain. As the axial strain continues to increase, the stress increment begins to decrease gradually and finally converges to a stable residual strength value. The phenomenon reveals the stabilization process of the internal structure and mechanical properties of the specimen after the plastic deformation stage. With the increase in *σ_z_*_0_, the stress increment of the specimen at the beginning of unloading increases significantly, indicating a larger initial modulus, and its peak and residual strengths increase significantly at the same time.

On the other hand, in the simulations with stress path B, the peak value of stress variation in the unloading direction (*σ_x_*) was lower than that of stress path A with the same initial stress state, because in stress path B, unloading takes place in the minor principal stress direction, and therefore it more easily reaches the critical state. In addition, shear dilation is observed during the whole process of the unloading triaxial test, which reflects their volume change characteristics during the unloading process. More significant shear dilation is observed in the simulation with stress path A rather than stress path B. In stress path A, shear dilation increases with the decreases in *σ_z_*, while no obvious difference can be observed in the volume strains of different triaxial unloading simulations that follow stress path B.

**Figure 8:**
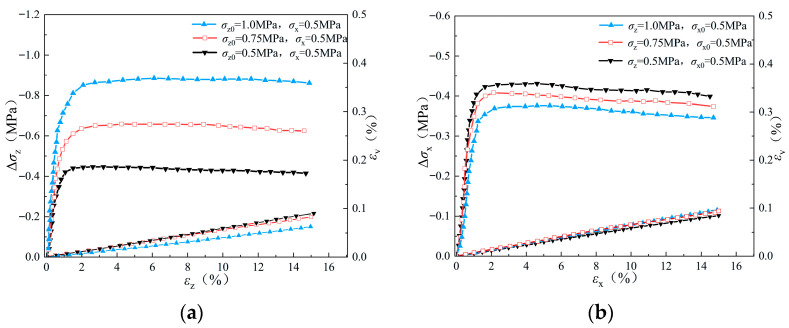
The evolution of stress–strain with axial strain during unloading of pebble sample (*e* = 0.47): (**a**) stress path A; (**b**) stress path B.

**Figure 9:**
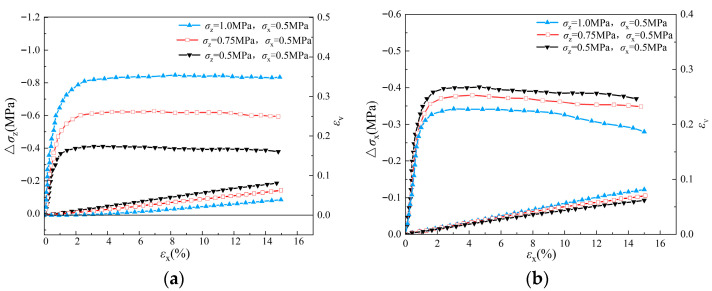
The evolution of stress–strain with axial strain during unloading of pebble sample (e = 0.67): (**a**) stress path A; (**b**) stress path B.

**Figure 10:**
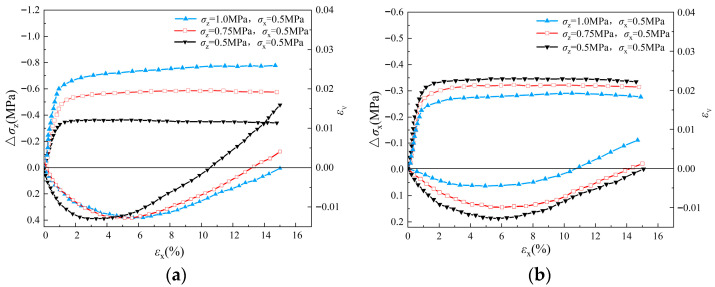
The evolution of stress–strain with axial strain during unloading of pebble sample (e = 0.81): (**a**) stress path A; (**b**) stress path B.

The stress–strain behavior of the pebble specimens under stress path A and B are presented in Figure 8, Figure 9 and Figure 10. Similar to the results of the sand specimen, with the increase in axial strain, the amount of stress increment increases gradually in the early stage of unloading. After reaching peak value, it begins to decrease to a relatively stable residual value with the continuous increases in axial strain. Under stress path A, both the peak and residual values of Δ*σ_z_* increase with initial *σ_z_*_0_, while under stress path B, both the peak and residual values of Δ*σ_x_* decrease with the confining pressure *σ_z_*. Compared to the simulation results of sand specimens, the peak and residual values of Δ*σ_z_* and Δ*σ_x_* of the pebble specimens under the same initial condition are much higher, indicating a larger shear strength of the pebble specimen. Therefore, under stress path B, the difference of Δ*σ_x_* in different simulations of pebble specimens is less than that in different simulations of sand specimens.

For the volume strain of the specimens, shear dilation is observed during the whole simulation in the dense sample (*e* = 0.47) and the middle dense sample (*e* = 0.67). However, in the case of the loose sample (*e* = 0.81), obvious shear shrinkage phenomenon occurs in unloading simulations. Under stress path A, shear dilatancy tends to be more significant with the decreases in Δ*σ_z_*_0_, while under stress path B, shear dilatancy in the simulation with larger Δ*σ_z_* is more significant.

### 4.2. Persistence Diagram

**Figure 11:**
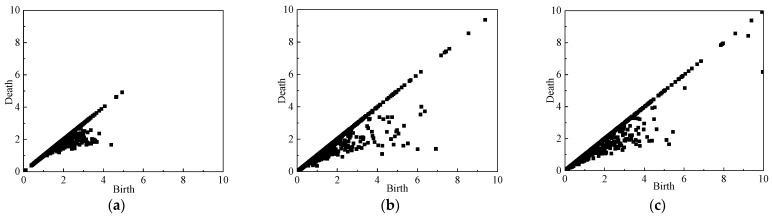
PD*β*_0_ of the pebble specimen (e = 0.47) at different axial strains: (**a**) *ε*_z_ = 0%; (**b**) *ε*_z_ = 2.4%; (**c**) *ε*_z_ = 16%.

**Figure 12:**
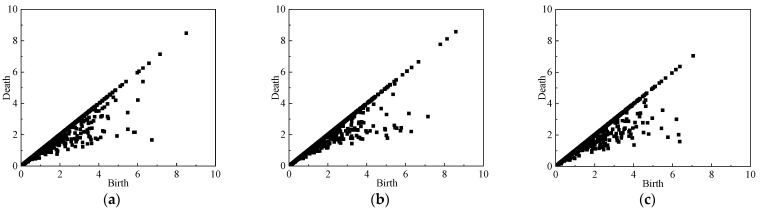
PD*β*_0_ of the pebble specimen (e = 0.81) at different axial strains: (**a**) *ε*_z_ = 0%; (**b**) *ε*_z_ = 2.4%; (**c**) *ε*_z_ = 16%.

**Figure 13:**
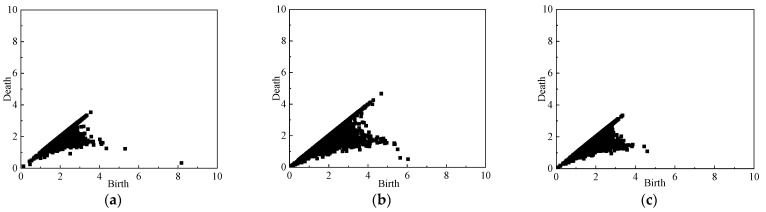
PD*β*_0_ of the sand specimen (*e* = 0.47) at different axial strains: (**a**) *ε*_z_ = 0%; (**b**) *ε*_z_ = 2.4%; (**c**) *ε*_z_ = 16%.

In order to analyze the mesoscale structures of the force network evolution during the whole unloading simulation, the persistence diagrams PD*β*_0_ of the normal contact force networks are computed. The PD*β*_0_ of the simulation with *σ_z_*_0_ = *σ_x_*_0_ = 0.5 Mpa at different stages during the unloading process obeying stress path A is shown in Figure 11, Figure 12 and Figure 13. Since *b* > *d*, the points in PD*β*_0_ are all located at the lower right of the diagonal line, and the points farther away from the diagonal line correspond to the more significant topological features in the contact force network, while the points closer to the diagonal line correspond to the shorter duration of network topological features, which have less influence on the overall network structure features. As can be seen from Figure 11a, in the initial state, the force network formed by the particles is uniformly arranged, and the horizontal and vertical coordinate values of the points in PD*β*_0_, *b*, *d* are smaller and closer to the diagonal. With the unloading process of the simulation test, the shear strain and stress changes gradually increase, the positional relationship between the particles is continuously adjusted, and the cluster-like structure with larger contact force gradually appears in the particle contact force network and develops gradually to maintain the stability of the overall particle assembly. Therefore, as shown in Figure 11b, compared with the initial state, the overall point cloud in PD*β*_0_ at the peak stress state (*U*_2_ state point) spreads in the direction away from the coordinate origin and diagonal. Thereafter, with further increase in shear strain, the cluster structure in the particle contact force network is gradually destroyed, and the peak stress of the particle assembly gradually decreases (See Figure 8). Therefore, as shown in Figure 11c, compared with the peak stress state (*U*_2_ state point), the overall point cloud in PD*β*_0_ at the end of the unloading simulation (*U*_5_ state point) shrinks again to the coordinate origin and the diagonal.

The PD*β*_0_’s of the loose pebble specimen (e = 0.81) during the simulation are shown in Figure 12. During the simulation process, the variation trend of the point cloud on the PD*β*_0_ of the granular specimen is basically the same as that in Figure 11. However, due to the small difference between peak stress and the residual stress in the stress–strain diagram of the loose specimen, the difference between the point clouds in PD*β*_0_ at the *U*_2_ state point and the *U*_5_ state point is also small.

Figure 13 shows the PD*β*_0_ of sandy soil particles. In the initial state, the horizontal and vertical coordinate values of the points in the PD*β*_0_ of sandy soil are small and relatively close to the diagonal. During the unloading process, the particles are arranged and the particle contact force networks are reorganized to gradually form clusters to maintain a relative stability of the structure of the force network, and the overall point cloud disperses outward. Then, the relatively stable force chains in the contact force network are gradually destroyed with the continuous increase in shear strain, resulting in the shrinking of the point cloud in PD*β*_0_. Comparing Figure 11 and Figure 13 of different particles under the same working conditions, coordinates b and d of the point in PD*β*_0_ of sandy soil are smaller than that in PD*β*_0_ of the pebble, because the pebble particle has a larger size so that the particle contact force between pebbles is larger than that between sand particles. In addition, the particle number of sandy soil specimens is much larger than that of pebble specimens, so the point cloud of sandy soil is much denser compared with that of pebbles.

### 4.3. Average Strength of Contact Force Network

From the above analysis of PD*β*_0_, it can be seen that the contact force network of soil samples with different densities shows different development patterns. In order to quantitatively reflect the variation in particle contact force network in different simulations, the concept of average strength of contact force network *F_ave_* [32] is introduced in this paper:

(1) $$F_{ave}=\sum_{i=0}^{n}(b-d)/N$$

where *F_ave_* is the average strength of the force chain, i is 1, 2, 3…N, b is the horizontal coordinate (birth value) of the points in PD*β*_0_, d is the vertical coordinate (death value) of the points in PD*β*_0_, and N is the number of data points in PD*β*_0_.

**Figure 14:**
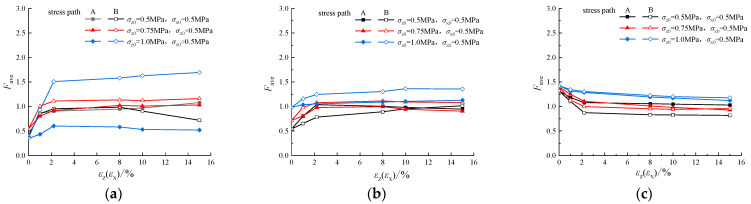
The average strength of force chain under different void ratios: (**a**) e = 0.47; (**b**) e = 0.67; (**c**) e = 0.81.

Variations in *F_ave_* in different simulations of the pebble with *σ_z_*_0_ = *σ_x_*_0_ = 0.5 Mpa are presented in Figure 14. According to Figure 14, in dense specimens (e = 0.47) and medium-density specimens (e = 0.67), the evolution of *F_ave_* has a similar trend with the stress–strain curve. In the initial stage, the dense specimens and the medium-density specimens are subjected to isotropic consolidation stress, leading to a uniform stress distribution inside the specimen. As a result, the force chain is effectively dispersed to each particle, and the average strength of the overall contact force network is relatively low. As the axial strain increases, the volume strain of specimens increases, the internal particles begin to rearrange and force chains with large particle contact force are formed in the contact force network. Therefore, the strength of the internal force chain is significantly enhanced. Then, after the specimen reaches the critical state, the internal particle contact force network structure gradually tends to stabilize, and the *F_ave_* also reaches a stable state.

However, in the loose specimen (e = 0.81) in Figure 14c, because of the large void ratio, most of the particles could not even form a strong force chain in the contact force network. The force network of the specimen undergoes natural fracture in the initial state. With the application of unloading stress, the network between the particles is further damaged, which ultimately leads to the gradual reduction in *F_ave_* in the specimen.

### 4.4. Particle Coordination Number

**Figure 15:**
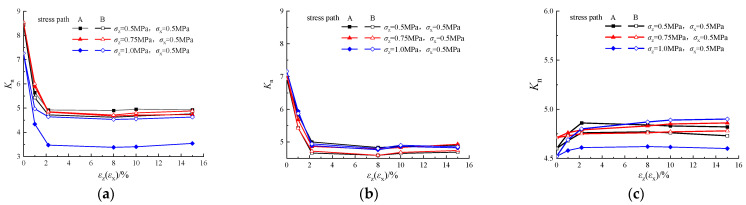
The relationship between average degree and axial strain during the unloading process: (**a**) compact specimens (e = 0.47); (**b**) medium-dense specimens (e = 0.67); (**c**) loose specimens (e = 0.81).

The particle coordination number is the average number of particles in contact with other particles in the granular material, and the average coordination number, also known as the average degree *K_n_*, is an important parameter to characterize the stability and pore space changes in granular specimens [35]. In this study, taking the pebble granular material as an example, the trends of the particle coordination number during the triaxial unloading simulation tests of each group are shown in Figure 15.

From the microscopic to macroscopic analytical perspective, Figure 15 demonstrates the internal microstructural changes in dense and medium-dense specimens when subjected to consolidation prestress. At the initial stage, the consolidation prestress causes the particles to be in close contact with each other, resulting in a higher average coordination number. This compact structure helps to reduce the porosity and enhance the stability of the specimens when carrying loads, reflecting the high averaging degree that characterizes the network, i.e., diverse connecting paths and efficient information transfer capability.

With the deformation of the specimen during unloading, the mutual friction between the particles increases, resulting in the particles starting to misalign, and the specimen gradually undergoes shear dilation, and the volume strain increases, which in turn leads to an increase in the porosity and a rapid decrease in the average number of coordination sites. However, as the specimen gradually reaches the critical state, the structure between the particles gradually stabilizes and the average coordination number stays at a constant level despite the continued increase in bulk strain. This indicates that the network maintains a certain connection density and structural stability even during the deformation process, and this stability is one of the important features possessed by networks with a high averaging degree.

In contrast, the initial porosity of the loose specimens was higher, and the average coordination number was lower. During unloading, as the specimen undergoes shear shrinkage, the porosity decreases and the average coordination number between particles gradually increases. Despite the decrease in volumetric strain of the specimen, the contact between the particles increased, thus contributing to the increase in the average coordination number. However, with further deformation and rearrangement of the specimen, the average coordination number eventually stabilizes, indicating that the internal structure of the specimen has reached an equilibrium state, and the relative position of the particles no longer changes significantly.

### 4.5. Discussion

In this work, multi-scale analysis on the responses of geotechnical granular materials under different unloading stress paths with different relative density was conducted through a series of DEM simulations of the triaxial unloading test. The main findings are summarized in Table 3.

As presented in Table 3, careful analysis of the contact force network structure through PH in this study provides new insight into the stress–strain behaviors of granular materials under unloading stress path. For dense and medium-dense samples, in the initial stage of shear strength development, particles in the sample rearrange with shear dilation, coordination number increases, and the particle contact force network is generated gradually with an increase in the average strength of the network to bear more external load. Then, as the volumetric strain continuously increases with shear dilation, the particle contact force network is destroyed gradually. As a result, the average strength of the contact force network decreases and the shear strength of the whole sample decreases, too. For the loose sample, because of shear shrinkage, the contact force network generated in the sample initially is destroyed during shearing. As a result, the average strength of the contact force network decreases and the coordination number increases, and strain hardening is observed on the stress–strain curve.

However, it should be noted that in this study, pebbles were modeled as clumped spheres to approximate their non-spherical shape, while sand grains were represented as single spheres, which is different than the real particle shape of granular soil. This simplified treatment may influence micromechanical behavior, particularly the formation and evolution of force chains. Specifically, the clumped-sphere representation for pebbles introduces a degree of interlocking and rotational resistance that single spheres cannot capture, which likely affects force chain stability and persistence. Based on previous studies that have examined particle shape effects on force chains (e.g., [16,22,23,36,37]), while the quantitative values of persistent homology metrics may vary with clump geometry, the general trends observed across different mixture proportions are likely to remain robust. This simplification represents a limitation of the current study and future work, such as photo-elastic tests, could systematically examine the sensitivity of persistent homology metrics to particle shape representation.

## 5. Conclusions

In this paper, the triaxial unloading triaxial test of sand and pebble specimens is simulated by the discrete element method, the behavior of the unloading state inside the granular material and the common characteristics of the force chain topological network are studied, and the fine structure differences under the same stress state during the unloading process are analyzed from the perspective of a complex network. The following conclusions are obtained:

(1) Dense bulk particles show strain softening and shear dilation during the triaxial unloading test, and in loose soil particle specimens, the body transformation will go through an obvious shear shrinkage phenomenon, and with the increase in axial strain, it slowly transforms to shear dilation. The peak strength and residual strength of the bulk particles are different under different stress paths.
(2) Based on the persistent homology theory, the contact between a large number of geotechnical particles is abstracted into a contact force chain network, and the analysis of the topological features of the granular materials can help to explain their mechanical properties. The changes in topological characteristics of the force chain network during the unloading process of soil particle specimens at different densities were investigated. The dense specimens exhibited rapid formation and annihilation of the force chain network after unloading, while the loose specimens showed a significant prolongation of the force chain life cycle. With increasing shear strain, the overall force chain structure of the specimen is gradually disrupted and reorganized, which is reflected in the diffusion and contraction of the point cloud on the sustained homography. This study reveals the relationship between the topological features of the force chain network and the initial state and strain degree of the specimens, providing important insights into mechanical behavior of particle aggregates.
(3) The micro-mechanisms of the internal structure evolution of the dense, medium-dense and loose specimens were revealed by analyzing the average strength of force chains and the average degree of granularity during the unloading process. The dense specimens showed high average strength of force chains and average particle size at the beginning of unloading, and then the force chain network was gradually destroyed and formed a cluster structure, and finally reached a stable state. The medium-dense specimens showed slightly lower average force chain strength than the dense specimens due to higher initial porosity, but their internal particle reorganization and stress adjustment processes were similar, and they also tended to stabilize. In contrast, the high initial porosity of the loose specimens resulted in lower average strength of the force chain and lower average degree of the particles, but the average degree gradually increased with the rearrangement of the particles during the unloading process.

## Figures and Tables

**Table 1:** Numerical simulation test group.

Material Type	Porosity	*σ_z_*/MPa	*σ_x_*/MPa	Stress Path
sand	0.35	0.5, 0.75, 1.0	0.5	A, B
pebble	0.35, 0.40, 0.45	0.5, 0.75, 1.0	0.5	A, B

**Table 2:** Mesoscopic parameter calibration results.

Soil Sample	Sandy Soil	Pebbles
Modulus value/*E*_*c*_ (GPa)	5.0 × 10^8^	2.0 × 10^9^
Normal stiffness/*k*_*n*_	5.0 × 10^9^	5.0 × 10^8^
Tangential stiffness/*k*_s_	2.5 × 10^9^	2.5 × 10^8^
Stiffness ratio *k*_*n*_/*k*_s_	2.0	2.0
Friction coefficient/*μ*	0.6	0.35

**Table 3:** Summary of the main analysis results.

Factors	Main Results
Stress–strain curve	(a) Obvious peak strength, strain softening and shear dilatancy of the sample with *e* = 0.47 and *e* = 0.67; (b) Strain hardening and shear shrinkage of the sample with *e* = 0.81; (c) The strength and shear dilatancy of the pebble sample are stronger than that of the sand sample under the same conditions;
Average strength of contact force network	(a) Increase in the average strength of the contact force network in the sample with *e* = 0.47 and *e* = 0.67; (b) Decrease in the average strength of the contact force network in the sample with *e* = 0.81;
Particle coordination number	(a) Decreases in the coordination number in the sample with *e* = 0.47 and *e* = 0.67; (b) Increases in the coordination number in the sample with *e* = 0.47 and *e* = 0.67.

## Data Availability

The original contributions presented in this study are included in the article. Further inquiries can be directed to the corresponding author.
